# The incidence and prevalence of diabetes in patients with serious mental illness in North West Wales: Two cohorts, 1875–1924 & 1994–2006 compared

**DOI:** 10.1186/1471-244X-8-67

**Published:** 2008-08-07

**Authors:** Joanna Le Noury, Afshan Khan, Margaret Harris, Winnie Wong, Dawn Williams, Tony Roberts, Richard Tranter, David Healy

**Affiliations:** 1 Department of Psychiatry, North West Wales NHS Trust, Bangor, Wales, LL57 2PW, UK; 2 Department of Psychological Medicine, Cardiff University, Bangor, Wales, LL57 2PW, UK

## Abstract

**Background:**

Against a background of interest in rates of diabetes in schizophrenia and related psychoses and claims that data from historical periods demonstrate a link that antedates modern antipsychotics, we sought to establish the rate of diabetes in first onset psychosis and subsequent prevalence in historical and contemporary cohorts.

**Methods:**

Analysis of two epidemiologically complete databases of individuals admitted for mental illness. 3170 individuals admitted to the North Wales Asylum between 1875–1924 and tracked over 18,486 patient years and 394 North West Wales first admissions for schizophrenia and related psychoses between 1994 and 2006 and tracked after treatment.

**Results:**

The prevalence of Type 2 diabetes among patients with psychoses at time of first admission in both historical and contemporary samples was 0%. The incidence of diabetes remained 0% in the historical sample throughout 15 years of follow-up but rose in the contemporary sample after 3, 5 and 6 years of treatment with an incidence rate double the expected population rate so that the 15 year prevalence is likely to be over 8%.

**Conclusion:**

No association was found between diabetes and serious mental illness, but there may be an association between diabetes and treatment.

## Background

A series of recent studies have uniformly shown a prevalence of diabetes in patients with schizophrenia 2 to 4 times greater than is found in the rest of the population [1-4]. Interest in a link between diabetes and serious mental illness has arisen against a background of concerns that antipsychotic agents might trigger diabetes. A competing hypothesis is that this increase in prevalence might arise if mental illnesses like schizophrenia predispose to diabetes. The evidence in favour of this latter hypothesis depends heavily on claims that the recognition of such a link antedates the use of antipsychotic drugs [3].

Current data suggests that between 3–4% of the population have diabetes [5,6]. If estimates that patients with serious mental illness are 2 to 4 times more likely to have diabetes than the rest of the population apply to an untreated population, while taking into account that diabetes has hugely increased in prevalence, we might expect a substantial number of those admitted to an asylum in the pre-antipsychotic era to have had manifestations of diabetes. We might also expect to find cases of diabetes among untreated first admissions for serious mental illness today. In North West Wales, we have historical and contemporary databases for serious mental illnesses that allow us to shed some light on these issues.

## Methods

There are a number of features of the datasets outlined below that offer opportunities to study the incidence of hospitalisation for and prevalence of hospitalised mental disorders and concomitant physical disorders. First, the population of North West Wales has remained essentially the same between the 1875–1924 period and now [7]. Second, geographical and financial constraints mean there has been nowhere else for patients with serious mental illness to access a service other than through the North Wales Asylum in the first instance and the services of the North West Wales Trust latterly. Third information systems in place enable us to track all patients and their care whether they remain in area or not. Fourth the population accessing services was and remains ethnically homogenous, with over 80% during the 1875–1924 period and over 66% in the contemporary sample having traditional Welsh surnames. For the purpose of this study we have looked at all admissions for schizophrenia, other non-affective psychoses, bipolar disorder, and manic episodes, as these are the patients who are now thought at risk of diabetes, and the patients most likely to be treated with antipsychotic agents.

### North West Wales historical database

The first dataset consists of admissions from North West Wales to the North Wales Asylum between 1875 and 1924. All diagnoses were made according to ICD-10 criteria before this study was undertaken [7]. The historical records offered four sets of information relevant to diagnosis of both mental illness and diabetes. First, all patients were compulsorily detained and their records included the medical and legal certificates outlining the circumstances that led to detention. Second, the records contain standard demographic data including age, sex, educational, employment and marital status, family history of illness and the individual's prior mental or physical illness. Third, there was a description of the patients' mental and physical state on admission, including reference to pre-existing disorders like diabetes, and prior mental illness. Fourth, there was a set of case notes covering the patient's stay in hospital until discharge or death, which in addition gave details of emerging physical illnesses and causes of death in patients who died in hospital. We could retrieve the records of prior admissions back to 1865 or subsequent admissions through to 1965. Clinicians making a diagnosis did so on the basis of a full set of records from all admissions for that patient rather than simply on the case record for that admission. We were also able to track the clinical course of patients for 50 years or more.

Based on this information, diagnoses for schizophrenia (F20), schizoaffective disorders (F25), delusional disorders (F22), acute and transient psychotic disorders (F23), and bipolar disorder (F31) were clear cut. A further group of patients were diagnosed as unspecified non-organic psychoses (F29), if the clinical details were insufficient to sustain a definitive diagnosis, or manic episode (F30) if the clinical course did not produce further episodes.

The presence of diabetes was readily determined from the notes, which distinguish clearly between diabetes mellitus and diabetes insipidus. Patients were noted to have diabetes mellitus on admission, or to have developed it while in care, or to have had the diagnosis recorded on their death certificates. Diabetes in this cohort connotes a medical diagnosis rather than a determination of diabetic status on the basis of glycaemic status or glucose tolerance test abnormalities. Once diagnosed, the condition could clearly be managed medically, with several patients surviving for over a year after first diagnosis.

We have established the incidence and prevalence of diabetes over 3, 5, 6 and 15 years from admission to compare the historical and contemporary cohorts. But we have also used the period of observation for the emergence of diabetes in the historical cohort, covering 13,117 hospital years and 18,486 patient years, to establish prevalence over the full treatment period.

### Contemporary first admissions for psychosis

The second dataset is drawn from an ongoing study of service utilisation for non-affective and affective psychoses from North West Wales. Patients were included in this study if they were native to or resident in North Wales following an initial hospitalisation for affective or non-affective psychosis. We have not included patients not resident in or native to North Wales, who had an initial illness episode in North Wales but left the region thereafter, such as college students.

From records of all patients admitted to the only District General Hospital Unit in the North West Wales, we have assembled all patients with first admissions between 1994 and 2006 for conditions with an F20 – F29 diagnosis or with a diagnosis or either bipolar disorder (F31) or manic episode (F30). Initial cross-sectional diagnoses made by sector consultant psychiatrists were supplemented by longitudinal data with renewed consultant and community mental health team diagnostic input.

The incidence of diabetes at first admission, and the emergence of cases within 3, 5 and 6 years of first admission, was determined by scrutinising the case notes in the first instance. Second we accessed the North West Wales hospital database for all patients listed in our cohort for evidence of diabetes not recorded in their mental health records. Third, the electronic records of all patients receiving hospital care for diabetes were scrutinised to access those with a coincident mental health diagnosis for possible cases of psychoses missed from the hospitalised cohort. Fourth we accessed pharmacy records for all patients receiving treatments for diabetes while in psychiatric inpatient care. Fifth we contacted the primary care physicians looking after all living patients in the cohort to establish whether any patients might be receiving treatment for diabetes who had not accessed the hospital medical services for diabetes treatment. A diagnosis of diabetes, in this sample, as in the historical cohort, connotes a medical diagnosis and the institution of management strategies, rather than a diagnosis based on inference from glycaemic or glucose tolerance abnormalities.

### Ethical approval

This study was approved by the North West Wales Ethics committee. Written informed consent was sought from patients in the contemporary sample not currently in contact with the mental health services to interrogate their medical histories.

## Results

### 1875–1924 cohort

In the period from 1875 to 1924, there were 3872 admissions from 3170 individuals to the asylum from North West Wales. In 39 cases incomplete records made psychiatric diagnosis impossible. Of the remaining 3833 admissions, there were 1,303 admissions from 1,040 individuals diagnosed with a psychotic disorder, of which 741 admissions were diagnosed with schizophrenia or schizoaffective disorder from a total of 593 individuals. A further 185 admissions from 153 individuals were diagnosed as delusional disorders, and 377 episodes from 294 individuals were diagnosed as acute and transient psychotic disorders or other non-organic psychoses. In addition there were 356 admissions from 135 individuals admitted for bipolar disorder and 74 admissions from 73 patients with manic episodes. There were therefore 1733 admissions from 1248 individuals in total. The comparative ages at first admission broken down by diagnosis of the historical and contemporary cohorts are laid out in Tables 1 &2.

**Table 1:** 1875–1924 Admissions by Age, Gender & Diagnosis

**Diagnosis**	**10–24**	**25–34**	**35–44**	**45–54**	**55–64**	**65+**	**Total**
**Schizophrenia**							
**Male**	79	113	64	26	8	5	295
**Female**	54	99	82	47	13	3	298
**Delusional Disorder**							
**Male**	-	6	15	22	10	10	63
**Female**	-	3	17	43	18	9	90
**Acute & Transient Psychoses F23**							
**Male**	20	12	18	10	3	7	70
**Female**	8	11	19	17	10	3	68
**Unspecified Psychoses F29**							
**Male**	19	19	16	11	12	3	80
**Female**	9	21	15	16	9	6	76
**Bipolar Disorder**							
**Male**	8	12	9	9	7	2	47
**Female**	12	26	22	17	5	6	88
**Manic Episode**							
**Male**	9	8	7	2	4	4	34
**Female**	17	9	9	4	-	-	39

**TOTAL**	135	170	129	80	44	31	**589**

**Table 2:** 1994–2006 Admissions by Age, Gender & Diagnosis

**Diagnosis**	**10–24**	**25–34**	**35–44**	**45–54**	**55–64**	**65+**	**Total**
**Schizophrenia**							
**Male**	65	41	15	3	-	-	124
**Female**	19	18	20	3	2	-	62
**Delusional Disorder**							
**Male**	1	4	5	2	6	4	22
**Female**	-	1	-	6	2	11	20
**Acute & Transient Psychoses F23**							
**Male**	7	8	4	1	2	1	23
**Female**	5	5	6	2	1	2	21
**Unspecified Psychoses F29**							
**Male**	21	19	3	2	1	1	47
**Female**	4	6	8	3	1	8	30
**Bipolar Disorder**							
**Male**	6	3	6	2	2	1	20
**Female**	3	9	7	2	1	3	25
Manic Episode*							
**Male**	-	-	-	-	-	-	-
**Female**	-	-	-	-	-	-	-

**TOTAL**	100	75	33	10	11	7	**236**
	31	39	41	16	7	24	**158**

*Manic episode patients included with Bipolar Disorder

At the time of admission, none of the 1248 patients with schizophrenia or other non-affective psychosis had diabetes. Two of the remaining 1972 individuals had diabetes on admission, one of whom was a 63-year-old man with chronic alcoholism. The second was a 48-year-old lady, who was recorded as having developed diabetes 12 months previously, who died 9 days after admission to hospital in a diabetic coma. The details of her pre-admission behaviour and clinical course point to an organic disorder rather than a psychotic disorder, as defined above.

A further 3 patients developed diabetes in hospital. Of these 2 patients came from the non-psychotic group – one had a learning disability and epilepsy and appears to have developed diabetes after 30 years in hospital. A second was a lady who was 58 years old on admission for severe recurrent psychotic depression who developed diabetes after 6 years in hospital.

The third patient was a lady who was 43 years old on admission with manic-depressive illness who developed diabetes after 30 years in hospital. This case gives a prevalence for the historical cohort of 1 case of diabetes in 1248 patients, followed closely for over 18,000 patient years. No case in the historical sample developed diabetes within 3, 5, 6 or 15 years of hospitalisation.

These 5 cases, two at admission, and three who developed diabetes subsequently, were admitted at regular intervals through the sample, from 1883 to 1923.

Diabetes prevalence is commonly cited separately for the 18–44 year old and for 45 years and older groups. Of the 1248 individuals with serious mental illness, 812 were in the 18–44 year age bracket at time of admission. At population prevalence rates for diabetes for the 1950s [5] assuming mental illness does not dispose to diabetes, we would have expected 3 cases from the 18–44 year age bracket and up to a further 8 from the older patients, 11 in total.

While the 1875–1924 prevalence of diabetes in the wider population was almost certainly lower than the prevalence estimates from the 1950s [4], it can be noted that a significant number of our sample lived through to the 1950s and the only case who developed diabetes from the target group was diagnosed in the 1950s.

### 1994–2006 cohort

There were 394 first admissions for schizophrenic, other non-affective psychoses, manic or bipolar disorders to the North West Wales mental health services in this period. Of these 186 had schizophrenia, 42 had delusional disorders, 44 had acute and transient psychoses, 77 had unspecified psychoses, and 45 had bipolar disorder or a manic episode.

One patient with an acute and transient psychosis had pre-existing Type 1 diabetes at the time of admission. No patients with schizophrenia or other non-affective psychoses, bipolar disorder or manic episode had Type 2 diabetes at the time of first admission (Table 3).

**Table 3:** Prevalence of Type 2 Diabetes in 1875–1924 & 1994–2006 Cohorts

**Diagnosis**	**Prevalence of Diabetes at Admission: 1875–1924**	**Six Year Prevalence of Diabetes: 1875–1924**	**Prevalence of Diabetes at Admission: 1994–2006**	**Six Year Prevalence of Diabetes: 1994–2006**
**Schizophrenia**	0%	0%	0%	3.3%
**Delusional Disorder**	0%	0%	0%	0%
**Acute & Transient Psychoses F23**	0%	0%	0%	0%
**Unspecified Psychoses F29**	0%	0%	0%	2.2%
**Bipolar Disorder**	0%	0%	0%	2.7%
**Manic Episode**	0%	0%	0%	0%

**TOTAL**	0%	0%	0%	3.1%

Six patients developed Type 2 diabetes, 5 males and 1 female, following their first admission. Of these 6, 4 have schizophrenia, one a psychosis linked to prior alcohol abuse (F29), and one bipolar disorder. Two of the 6 developed diabetes within 3 years of first hospitalisation for serious mental illness, both under the age of 45. Two more developed diabetes within 5 years, one under 45 and one over 45. A further two developed diabetes shortly after 5 years of follow-up, one under 45 and one over 45. All 4 patients under the age of 45 were on ongoing treatment with atypical antipsychotics.

Within this contemporary sample, 319 were under the age of 45 at point of first admission. Population norms for the contemporary prevalence of diabetes suggest we might have expected 3 patients with diabetes in this cohort.^5 ^There were a further 75 patients over the age of 45 at time of first presentation, from whom 4.5 cases of diabetes might have been expected. The expected number of diabetes cases therefore was 7, whereas the observed prevalence on first admission was 1 case. The expected number of cases after 6 years of follow up in those under 45 was 3, whereas the observed number was 5 cases.

As of the time of writing, 1.5% of the original contemporary cohort has Type 2 diabetes. However, 36 of the cohort have died and not all the cohort has 3, 5 or 6-year follow up data. Basing prevalence estimates on the proportion of living cases with completed data in each of these follow-up periods, the prevalence of diabetes at 3 years was 0.7%, at 5 years was 1.8% and at 6 years was 3.1%. This 6-year figure gives an incidence rate of 5.1 cases/1,000 patient years. At 15 years the prevalence rate is likely to be over 8%.

When patients with acute and transient psychoses, and manic episodes, as well as bipolar disorders are removed, the prevalence of diabetes in non-affective psychoses (primarily schizophrenia) is 0.9% at 3 years, 2.4% at 5 years, and 3.3% at 6 years. This latter figure gives an incidence rate of 5.4 cases/1,000 patient years.

Comparing the historical and contemporary cohorts, the relative risk of presenting at first admission with Type 2 diabetes for both cohorts was similar. While any comparisons between the relative risk of developing diabetes for the contemporary and historical cohorts are necessarily compromised by the different prevalence of the disorder during the two periods, our data at present point to a 0% 15 year prevalence in the historical cohort compared with a likely 8% prevalence in the contemporary cohort. Based on actual cases at any time point, the data points to a 21.9 times (95% C.I 2.7, 177.8) greater risk for the contemporary cohort.

Including cases from the index cohort, our interrogation of general hospital, pharmacy, and general practice records netted 71 patients with diabetes and a non-affective disorder psychosis or bipolar disorder that have had hospital contact. There were 1031 individuals listed in the electronic database of the North West Wales Trust as having received medical care between 1996 and 2006 who had a diagnosis of both diabetes and a mental illness. Of these 1031 individuals, 368 had a dementing or other organic disorder (F00–09), of whom a large number died within months of diagnosis, 204 had an alcohol or other substance abuse related disorder (F10–19), 45 had a non-affective psychotic disorder (F20–29), 230 had an affective disorder (F30–39) and 284 had an anxiety based or personality linked problem (F40–69).

## Discussion

The figure reported here for 6-year prevalence for Type 2 diabetes in the contemporary cohort is over 3%. This is not inconsistent with the 13% figure for prevalence of diabetes in a sample of living patients after an average of over 14 years of treatment for schizophrenia stemming from the recent CATIE study [2]. Our findings show an incidence rate of diabetes in the contemporary sample of schizophrenic patients of 5.4/1000 patients per year, which is double the figures for the incidence of diabetes for the population in general of 2.4/1000 patients per year [5]. This puts us on track for a 15-year prevalence rate of 8%; the final figure may be greater as diabetes incidence rates increase with age [4].

This incidence rate is lower than incidence rates of 1.3% and 4.4% reported for other groups of psychotic patients [8,9]. These other samples however have not been drawn from patients newly admitted for psychosis and the mean age of the samples and mean duration of treatment have almost certainly been greater than the mean age and duration of treatment found in this sample.

As a control on our findings, we report data from the combined medical and psychiatric services for the prevalence of diabetes in subjects with any mental illness. These are consistent with findings for diabetes in general – it is most common in the elderly (dementia) and in women and in those with substance misuse problems – suggesting that our "assay" system has been performing acceptably.

There are, nevertheless, a number of limitations to this study. In the first place it is a study of service contacts only rather than a complete survey of all incident cases of either psychosis or diabetes in the wider population. Second, a number of health service populations are put on antipsychotics, including cases of dementia, alcohol abuse and personality linked problems, but we have not sought to establish the prevalence for diabetes among all patients before and after treatment with antipsychotic drugs. Third we have not sought to determine the prevalence of diabetes among previously hospitalised chronic psychotic patients, as many of these are managed exclusively in primary care, thus creating problems in ensuring we have a complete sample. Fourth, our definition of diabetes is a clinical one: we have not sought to determine on the basis of blood glucose values all possible cases of diabetes. Fifth owing to changing population prevalence for diabetes, the historical and contemporary cohorts cannot be directly compared.

However, the study is epidemiologically complete, in that we can be reasonably certain there is no one from this catchment population of 240,000 during either the historical or contemporary periods who had a first episode of psychosis and who accessed services of whom we are unaware. Second of those receiving a service, we can be reasonably confident that we have detected in both cohorts all cases receiving a clinical diagnosis of diabetes. Third, the study sheds light on the incidence rate for diabetes in a first admitted population. Fourth, this study uniquely sheds light on an epidemiologically complete population from the pre-antipsychotic era.

The findings are inconsistent with widely cited suggestions of a link between diabetes and serious mental illness. The present study suggests strongly that patients with serious mental illness are not predisposed to diabetes. These findings are consistent with recent studies that find no evidence of diabetes at the point of first admission for schizophrenia [10,11]. Indeed the findings are not inconsistent with the possibility that untreated serious mental illness has a protective effect against diabetes.

Current perceptions of a link between serious mental illness and diabetes stem in part from a series of articles from the pre-antipsychotic. A great deal hinges on claims that Henry Maudsley had anecdotally noted a connection between mental illness and diabetes in the late 19^th ^century. Aside from this claim there is in fact no other reference to patients having diabetes.

A series of articles have reported hyperglycemia or abnormal glucose tolerance tests in patients with serious mental illness [12-17]. It is not clear however that these findings should be taken as indicators of unrecognised diabetes. Much of this early work was undertaken on catatonic patients, who it is well known demonstrate autonomic instability leading to fluctuations of blood pressure, temperature and heart rate, which normalise on recovery. Just as one would not take blood pressure elevations in catatonic states as evidence of latent hypertension, it may also be a mistake to interpret fluctuating glucose levels or abnormal glucose tolerance tests in untreated mental illness as indicative of a pre-diabetic state. The authors in these papers do not describe any of their patients as diabetic.

In contrast to the relative lack of data supporting a link between diabetes and serious mental illness, from as early as the mid-1950s, a series of articles have outlined elevations in glucose levels or aggravation of pre-existing diabetes following treatment with the earliest antipsychotics [18-23].

While Type 1 diabetes with an onset in childhood was inconsistent with survival before 1924 and therefore such cases could not have presented to the asylum, Type 1 cases remain rare today, and recent population studies suggest there is no correlation between Type 1 diabetes and psychosis [12].

Type 2 diabetes is the only kind that could have led to suggestions of a link between the two illnesses and, today, Type 2 accounts for 85% of cases of diabetes. Type 2 diabetes was readily recognised in the historical period and managed by dietary measures, with patients often surviving for years. The data from the historical period in this study contrast with the regularly cited anecdotal observation from Maudsley, a man who had very little exposure to patients, about a link between mental illness and diabetes. Indeed one value of this study is that it indicates how unreliable the observations of a single clinician are likely to have been, particularly during an era when diabetes was less prevalent than now.

While factors such as lifestyle and diet unquestionably play a part in producing the differences between contemporary and historical cohorts, in this case the contemporary cohort can act as their own control for such factors, and it seems unlikely that changes in lifestyle alone would produce a doubling of the expected incidence of diabetes in patients in such a short period of time, without the operation of a further contributing factor, such as drug treatment. A further pointer to the role of drug treatment lies in the distribution of incipient cases of diabetes which were more likely to arise in the schizophrenia than from other groups in the contemporary cohort. While compliance with ongoing therapy cannot be assumed in this group, it nevertheless seems more likely in this group than in the patients with acute and transient disorders for instance, who are encouraged to discontinue treatment on recovery.

## Conclusion

The absence of cases of Type 2 diabetes before the onset of psychosis, in either the contemporary or historical mental illness samples, combined with a more rapid rate of development of Type 2 diabetes in the contemporary sample than is the norm in the population in general, appears to make it more likely that treatment with antipsychotic drugs rather than the underlying illness contributes to the current prevalence rates found for diabetes in patients with serious mental illness.

## Competing interests

JLN none; AK none; MH none; WW none; DW none; TR has had support to attend meetings from Lilly, Bristol-Myers Squibb, and Janssen; RT has had support to attend meetings from Lilly, Astra Zeneca, Wyeth and Janssen; DH has had consultancies with, been a clinical trialist for, been a speaker at symposia for, or received support to attend meetings from Astra-Zeneca, Boots/Knoll, Eli Lilly, Janssen-Cilag, Lorex-Synthelabo, Lundbeck, Organon, Pharmacia and Upjohn, Pierre-Fabre, Pfizer, Rhone-Poulenc Rorer, Roche, SmithKline Beecham, and Solvay-Duphar. He has been expert witness for the plaintiff in 15 legal actions involving SSRIs and one patent case involving an antipsychotic.

## Authors' contributions

All authors contributed to the collection and analysis of the data. DW was on maternity leave while the manuscript was drafted.

## Pre-publication history

The pre-publication history for this paper can be accessed here:


